# Student perspectives of extended clinical placements in optometry: a qualitative study

**DOI:** 10.1186/s12909-022-03132-0

**Published:** 2022-01-25

**Authors:** Jacqueline M. Kirkman, Sharon A. Bentley, James A. Armitage, Ryan J. Wood-Bradley, Craig A. Woods

**Affiliations:** 1 Deakin Optometry, School of Medicine, Deakin University, Waurn Ponds, Australia; 2 School of Optometry and Vision Science, Queensland University of Technology, Kelvin Grove, Australia; 3 School of Optometry and Vision Sciences, University of New South Wales, Sydney, Australia

**Keywords:** Clinical placements, Longitudinal integrated clerkship, Students, Supervisors, Training, Education

## Abstract

**Background:**

The number of students enrolled in health courses at Australian universities is rising, increasing demand for clinical placements. Optometry students have historically undertaken clinical training in short-block rotations at university-led teaching clinics in metropolitan locations. This is changing, with some optometry programs adopting extended placements. These placements are conducted in community-based practices, with many incorporating a rural component to the training. This study explored factors which influence placement success and satisfaction from the perspective of optometry students.

**Methods:**

Nine focus groups were undertaken with 42 final year optometry students upon completion of a 26-week placement (of which at least half was undertaken in a non-metropolitan area, or area where a shortage of optometrists has been identified). Focus groups were audio recorded and transcribed verbatim. Thematic analysis was conducted according to Braun and Clarke’s 6 step method.

**Results:**

Four key themes were identified during analysis. ‘Changing identity’, related to how the students grew both personally and professionally, with the extended placement being considered the vital component that allowed students to begin thinking of themselves as clinicians. The theme ‘Dealing with complex dynamics and circumstances’ predominantly described instances where the student-supervisor relationship was strained, resulting in high levels of anxiety made worse by a perceived lack of university support. ‘Optometrist under instruction’, related to students feeling that the placement was an ideal opportunity to trial the everyday reality of work without the obligation of an ongoing commitment or employment contract. Finally, the theme ‘Rural practice is more rewarding’, was about a chance to seek different experiences, meet new people and challenge themselves professionally.

**Conclusion:**

While the majority of students enjoyed their placement and felt that it was the key component of their training that equipped them for future practice, it is clear that universities and placement providers must provide both students and supervisors thorough and explicit guidance covering placement expectations. Furthermore, student support systems should be embedded into placement programs to ensure where issues arise, they are dealt with promptly and successfully. It is vital that ongoing professional development and pedagogical training for supervisors underpins continued accreditation.

**Supplementary Information:**

The online version contains supplementary material available at 10.1186/s12909-022-03132-0.

## Background

Clinical placements during a student’s education are regarded as critical to achieving clinical skill proficiency, confidence and professional behaviour [1, 2]. Traditionally, clinical training of medical, nursing and allied-health students in Australia has occurred in public hospitals or university-led teaching facilities in metropolitan locations [1, 3]. These placements have frequently been conducted in short-block rotations [3]. As the number of students in health courses is rising, Universities and clinical education providers look for alternative methods of student placement [4]. Furthermore, global policy recommendations suggest student should be trained closer to rural communities in order to improve the maldistribution of the health workforce, which is currently biased towards practice in urban localities [5]. There is a growing body of evidence criticizing short placements, predominantly because they are seen to be difficult learning environments for students [1]. Their transient nature may mean students are unable to participate wholly in patient care [2]. Likewise, frequent changes in clinical supervisors can lead to students struggling with different supervisor styles and expectations [1]. Moreover, due to time constraints, students may not have an opportunity to foster social and spatial connectedness to the rural community in which they are placed, which is important in rural practice [6].

In medicine, Longitudinal Integrated Clerkships (LICs) “represent a transformative approach to clinical education that uses continuity and relationships among medical students, patients and physicians to shape the educational experience” [7]. The Consortium of Longitudinal Integrated Clerkships characterises LICs as having the following three core elements which include medical students: participating in the comprehensive care of patients over time; have continuing learning relationships with these patients’ clinicians; and meeting, through these experiences, the majority of the year’s core clinical competencies across multiple disciplines simultaneously [8]. Originally established to address a number of workforce and health system concerns [9], LICs have also addressed some of the issues associated with short placements [1, 10]. In particular, their focus is on the capacity for students to experience continuity [11]. Continuity has been described in terms of: patient care, which enables students to build trust and a professional relationship with their patients; geographical continuity, which allows students to access information from electronic records and achieve diagnostic closure and learn from their decisions; and supervisor continuity, which allows mutual confidence to be built between student and supervisor [1, 2, 10, 11]. LICs have been shown to create an increased sense of belonging and the development of collegial relationships between the student and supervisor [12]. Furthermore, academic performance during LICs is equal to academic performance in traditional rotational based clerkships [13, 14]. While not always, LICs are often based in non-metropolitan locations and as such give students a chance to experience life in a rural setting. LICs can be a means for academic institutions to accommodate increasing numbers of students [12]. However, concerns related to LICs include; lack of variability in clinical exposure, increased financial burdens for students, increased burden for clinical supervisors, and increased administrative burden for academic clinical coordinators [1].

There is great diversity in implementation of LICs across programs. Worley et al. [7], found that lengths of clerkships being described as LICs varied from 6 to 54 weeks during their study aimed at establishing a baseline reference typology for LIC programs. In that study, Worley and colleagues delineated and sorted existing LICs into three major groups based on whether they fit the definition of a conventional LIC, a hybrid LIC or an emerging form of LIC [7]. Theoretical educational frameworks suggest experiential [15] and work-based learning [16] are core in community-based placements such as LICs [17]. Learning in a situated clinical environment provides not only a real life, relevant clinical context in which learning can occur, but also authentic interactions that are fundamental for experiential learning [15]. The everyday engagement in work means there is generally no separation between participation in work and learning [16]. Extended clinical placements, modelled on the LICs used in medical training, have been adopted by some Australian optometry programs [18]. As optometry in Australia is not a profession with speciality disciplines, these extended placements should not be described as LICs as the third core element (as described by the Consortium of LICs) is not directly applicable. Instead, extended placements in optometry can be considered most similar to the Longitudinal Placements described by Harding in which UK based medical students are overwhelmingly sited in general practice [19]. In optometry, placements involve students delivering care under the supervision of an optometrist in a community practice for a continuous period of time (typically for between four to 26 weeks, at least four days a week) towards the end of their training [18]. Therefore, while different to traditional LICs, such placements may still offer the kind of structure which allows relationships to be built between student, patient and supervisor, and for longer-term patient follow up to occur [19].

Although there has been research focusing on medical and nursing student education, there is a dearth of studies investigating clinical placements in the optometry profession. Given the differences in scope of practice and employment settings, as well as the clinical supervisory models and resourcing available to medical and nursing programs, results from these disciplines may not be so easily transferable to other professions [20]. For instance, extended placements in optometry are undertaken in independent practices, not tertiary hospital settings, and supervisors are not paid or financially reimbursed for hosting a student. Moreover, the student is usually the only student at a placement site and there are no placement coordinators from outside the university. This study aimed to develop an understanding of the experience of extended clinical placements from the perspective of final year optometry students. In particular, the study aimed to describe factors which influence placement success, including whether there is a perceived difference between urban and rural placements.

## Methods

The study adhered to the tenets of the Declaration of Helsinki and was approved by the Deakin University Human Research and Ethics Committee (HEAG-H 1422017).

### Study design and philosophical basis

A qualitative descriptive approach was taken in this study [21–24]. This approach was considered appropriate as the views of optometry students have not previously been investigated and the authors sought to maintain a broad analytic scope thereby allowing the potential for the development of truly innovative themes [22, 24]. The aim of this study was to provide a rich, straight description of the participants experience rather than the development of theory or interpretive meaning [25]. The origins of qualitative description are based in naturalistic inquiry principles [24, 26]. As such, the world can be seen as subjective, with multiple realities which are experienced differently by each individual [24]. Qualitative description is focused on discovering the ‘who, what and where of experiences and gaining insights from informants regarding poorly understood phenomenon [26]. Ultimately, the qualitative descriptive approach was deemed particularly fitting as the authors sought to ‘stay close to and describe the participants’ experiences’ [24, 25].

### Context and participants

This Australian study sought the views of students from Deakin University’s combined Bachelor of Vision Science/Master of Optometry degree. The degree was established in 2012, in part to address the shortage of optometrists in regional and rural Australia. The degree is delivered in ten consecutive trimesters enabling a program that would traditionally take 5 years to deliver with a traditional semester series, to be completed in three and half years: The Bachelor component, in the first six trimesters, focuses on acquisition of vision science and biomedical knowledge, clinical skills techniques and clinical diagnostic thinking; The Masters component, in the final four trimesters, continues with advanced vision science and biomedical knowledge acquisition while providing opportunities for students to apply this knowledge over a range of clinical placements, culminating in a compulsory extended placement in the final two trimesters of the Masters portion of the combined degree. This extended placement is 26 weeks (2 × 13 weeks), and since 2016 at least 13 weeks must be completed in a designated non-metropolitan setting or area where a shortage of optometrists has been previously reported [27]. The vast majority of these placements took place in accredited private community practices in Australia. A small number (< 5%) were undertaken in New Zealand. To maintain participant confidentiality, the geographic locations in which students undertook their placement has not been reported in detail in this study.

Practice accreditation involved practices being able to show that they had sufficient room for the student to see patients under supervision, and diagnostic equipment required to practice full-scope optometry. Supervisors were required to have at least 3 year’s experience post-graduation, and at least one of the supervisory members needed to be endorsed to prescribe therapeutic agents. Accreditation occurred only after completion of a supervisor training course (which took approximately 4–6 h to complete).

All 152 Deakin Optometry final year students of the graduating cohorts of 2018 and 2019 were invited to participate in focus groups.

### Data collection

Group processes associated with focus group discussions were hoped to motivate participants to speak up explore their views, producing rich data [21]. Participation was voluntary and all participants provided informed consent. The focus groups occurred following the students’ extended clinical placement in May 2018 and May 2019. Nine focus groups with between 4 to 5 participants were conducted face-to-face by the first author and ran for approximately 60 min. A semi-structured interview guide was developed by the authors following an initial literature review that included relevant publications surrounding student placement experiences. This interview guide consisted of core open-ended questions with additional prompts to elucidate further detail (Additional file 1: Appendices 1). Lines of questioning focused on:

1. The positives and negatives of the placement.
2. Whether the students felt prepared for the placement.
3. How the placement could have been improved.
4. Whether the rural placement differed from the urban placement.

Each focus group was audio-recorded before being transcribed verbatim by an external agency (Pacific Transcription, QLD Australia).

### Analysis

Data were analysed using inductive thematic analysis. Analysis occurred in Nvivo Pro software, Version 11 (QSR International Pty Ltd., Doncaster, VIC Australia) independently following the principles of thematic analysis according to Braun and Clarke’s 6-step method [28] by two researchers, one optometrist (the first author) and one non-optometrist (the research assistant). Each transcript was audio-checked by the first author and research assistant for accuracy and to ensure data immersion occurred as part of Step 1. During this and the subsequent step the two researchers independently generated lists of initial ideas about what appeared interesting in the data. Throughout Step 2 initial codes were generated from the data, with segments of text that held the same meaning being labelled and collated together [29]. Step 3 involved grouping codes into categories where patterns and key aspects of interest were identified and combined to form themes. During this and Step 4, mind-maps were utilised to sort the different codes into themes. In step 3, 4 and 5, the two researchers met and themes were described, defined and revised to ensure data within the themes were cohesive, captured the significance and substance of the entire coded data set and there were identifiable distinctions between themes [30]. Researcher consensus was required in some instances, with differences in coding and theme identification being resolved by discussion between the two researchers. Further discussion and debate surrounding theme identification was undertaken with the rest of the research team before the final set of overarching themes and final report was generated in Step 6. This brought modifications of the naming of some themes. Participants’ language was used in all levels of coding and theme development to ensure that participants’ voices were clearly articulated. These steps were not necessarily undertaken in a linear fashion but rather, were recursive in nature.

### Trustworthiness

A number of techniques were utilised to ensure methodological rigor [21, 23, 30, 31] specific to qualitative description [23]. Credibility was established through rapport, trust and empathy [23]. Focus groups were conducted by the first author, a recently graduated optometrist trained in qualitative techniques and interviewing. Having a recent graduate interview final year students ensured the interviewer understood the terminology and culture within which the interviews took place, making it easier for appropriate follow up questions to be asked. It was also hoped students would find it easier to relate and build rapport with a recent graduate and thereby show greater willingness to exchange information [23]. A welcome introduction was read out by the interviewer at the beginning of each focus group which thanked participants for their involvement, reminded them of the confidential nature of their participation and explicitly encouraged all views to be shared. It was hoped this would provide a sense of mutual respect and trust [23].

The first author kept detailed notes and an audit trail to ensure credibility, dependability and confirmability of the results [23, 31]. Notes were used to record data collection and analysis decisions as well as the first authors feelings, thoughts and questions during analysis. These reflexive notes allowed the first author to consider how their background and views may potentially influence analysis.

Similarly, to allow greater dimensionality, analysis was conducted by two researchers with divergent backgrounds and disciplinary expertise [30]; the first author, a recent optometry graduate who had personal experience of a similar placement to that undertaken by the participants; and the research assistant, who had no experience of student placements in a healthcare setting and a background in international relations and research. The two researchers regularly met to discuss the data and how their own background and experiences influenced their interpretation, ultimately adding to their understanding and producing joint interpretation [32]. Finally, to provide transferability, the context, location, people studied are described in detail [31].

## Results

### Characteristics of participants

Forty-two students participated in this study. The majority identified as female (28 of 42, 67%). To give context to the participants statements generally, the geographic location they identified as having lived the longest prior to university study is illustrated in Table 1. In terms of their placement location; 11 students (26%) undertook both of their 13 week placements in locality 3–6 (see Table 1 for definition of locality); 26 students (62%) spent 13 weeks in a major metropolitan area (locality 1) and 13 weeks in a regional or rural location (locality 3–6); and the remaining students spent 13 weeks in a major metropolitan area (locality 1) and 13 weeks in locality 2 where a shortage of optometrists has previously been identified [27].

**Table 1:** Geographic location lived the longest (i.e., their hometown prior to university study)

Geographic area (population size)	Participants
Locality 1: Large Capital City (> 350,000)	(20 of 42, 48%)
Locality 2: Outer Metropolitan area or Small Capital City (100,000 to 349,999)	(11 of 42, 26%)
Locality 3: Large Regional Centre (25,000 to 99,000)	(4 of 42, 9%),
Locality 4: Small Regional Centre (10,000 to 24,999)	(1 of 42, 2%)
Locality 5: Rural Area (5000 to 10,000)	(4 of 42, 9%)
Locality 6: Remote Area (< 5000)	(2 of 42, 5%).

### Themes

Four main unifying themes and eleven subthemes were identified and described during analysis (Table 2). Alongside their corresponding participant number, quotes from participants are presented to further illustrate the findings.

**Table 2:** Themes and subthemes: Student perspectives

Theme	Subthemes
Theme 1: Changing identity	• Moving from student to clinician • Personal development and growth
Theme 2: Dealing with complex dynamics and circumstances	• Power dynamics and managing relationships • Lacking an advocate • Expectations not met • Lack of control, burnout and burdens
Theme 3: Optometrist under instruction	• Complete immersion and trial without commitment • Needing a mentor • Fear and uncertainty
Theme 4: Rural practice is more rewarding	• Rite of passage • Lack of ophthalmology support • The drive to return home

#### Theme 1: Changing identity

The students spoke enthusiastically about the extended placement being the vital component which allowed them to transition from student to clinician. They discussed coming to the realisation that real-world clinical practice often differed to textbook examples. They felt that their study at university gave them the foundational skills but that the placement truly enabled them to begin thinking of themselves as an optometrist. The extended duration of the placement was perceived as the key factor.

P51 “I think the first three months was more about just polishing skills, trying to figure out how to see a full patient consult….the next three months it was more consolidating it all…...the finishing touches I suppose….by the end, it was more that the supervisor was just there for formality. You didn’t actually need them to teach you anything new.”

The students described the length of the placement as appropriate.

P32 (the placement length was) “just about right. I wouldn’t want to do anymore, but I’m glad I didn’t do any less.”

The majority of students described an initial phase of apprehension and uncertainty while they ‘settled in’. A sense of comfort came with the continuity offered by the extended placements. Students detailed progressing from feeling like an outsider to an accepted member of the team. The duration enabled them to develop a good working relationship with staff and in particular, their supervisor. Students felt that they were of more help to the practices because they were there for a longer period of time, increasing the sense that they were a legitimate asset to the practice.

P34 “You start building relationships….And you’re like “I could fit in here.” Whereas maybe if you’re only there for a week or two, you wouldn’t have that”.

Many of the students described how remaining in one location for a longer period of time enabled them to become immersed in the community. This was particularly evident during their rural placement, where they recounted how their experiences gave them a greater sense of connectedness to the community.

P36 “Coming to a smaller town I know when I moved, within weeks I had several optometrists who have embraced me and taken me out.….I don’t think I would get that if I was working in a city”.

Students felt their rural placements offered them the greatest learning opportunity as they described seeing a greater diversity in patients, pathology and exposure to therapeutic prescribing. The majority suggested that it was during their rural placements where they experienced the most clinical growth.

P48 “My two placements were quite different, in pathology-wise and experience. One of my placements was like right in the CBD, and it was a lot of just…people just coming in for glasses. It was a lot of the same stuff. But then in my other one, it was more rural, and I found there was a lot more pathology…there was a lot more therapeutics. So, it was probably a better experience in terms of pathology-wise. It made it a bit more interesting…..”

For a number of the students, their time on placement was their first experience living independently. While this was initially anxiety-provoking, they expressed feeling safe to extend themselves as there was comfort in knowing that they were only committed for a relatively short period of time. Some students used the placement as a time to try something new. For a few students, it was also their first exposure to adult working life.

P55 “I’ve always been at home and I’m pretty sheltered…So I was like ‘Oh this is my opportunity to go somewhere else where I’ve never been before.”

#### Theme 2: Dealing with complex dynamics and circumstances

Students repeatedly emphasised the supervisor-student relationship as being the single most important factor in their placement experience. Although the majority of students had a good working relationship with their supervisor, some described circumstances where personality clashes, miscommunications, bullying, and boundary crossing occurred.

P52 “I think it’s very supervisor-dependent. My supervisor didn’t like taking students, straightaway didn’t like me, didn’t give me a chance. Every day, I went in with anxiety and came home crying……The hardest part about placement was managing that relationship.”

A few students described having supervisors who seemed to be unsure about how to approach supervision and appeared resentful of the student’s presence, treating them as if they were an inconvenience.

P37 “I do think that not everyone can be a teacher…...”

They expressed their unease during circumstances where they felt they were being used as a member of staff or as a personal assistant, to the detriment of their learning, and without financial reimbursement. Students perceived there to be a large power differential between them and the supervisor.

P76 “If you've got bullying for example, yes the protocol is you talk out about it, and you raise your hand, go, "Look, this is not right." But you've actually got to take the courage to go, "Yeah it's not right," but at the same time you could get all the backlash. And you've got to face that as well. You've got to be willing to face that. And I think weighing that up, like the backlash versus getting through this course, and not wanting to repeat another six months again, I felt that was a more important factor, to just go through it, just take it. Because it's only for three months.”

In the majority of instances, students chose not to raise issues with the supervisor or advise the University of their concerns. This was because they feared reprisal, being seen as a troublemaker or that word would spread to potential future employers of them being ‘difficult’. Instead, they described ‘dealing with it’ as they felt they could ignore and/or cope with the difficult circumstances in the time they had remaining on placement.

P58 “I was afraid to speak up and challenge them because they’ve got the power to mark us down and make an issue for me with the Uni as well. So in the end, I had to just kind of put my head down and just do what they said.”

In some instances, the students did seek assistance from the University to resolve concerns but were not satisfied with the response. Some students felt that the University valued the relationship with the supervisors more than the students.

P38 “I had a problem so I approached them (the University). I approached them so many times. But they were sort of ignorant to what I was saying. They sort of just—rubbed me off—said that I’d just have to get by.”

However, this was in contrast to the experience of other students who did feel supported by the University when they experienced issues while on placement.

P39 “Deakin was incredibly supportive. They were my advocates….they really supported me.”

Relocation and transport costs were particularly challenging for students who were not able to attend placement where they had existing networks. For many students, relocation for placement resulted in loss of employment income. Students described the stress which came from reduced earnings, particularly where they had existing financial commitments, such as mortgages and partner considerations. Often, they were not able to return home to visit family and friends during placement due to the cost. Students described wanting the experience of rural remote placement, but that the associated expenses were too much of a disincentive.

P34 “placement was really hard financially and I didn’t get to go home…. So being away from home, without being able to work, that was really hard.”

P39 “I would have loved to have gone more remote, but I couldn’t afford it.”

The availability of accommodation was location dependent. Where students had difficulty, they lamented that they had limited support from the University. In some circumstances students were required to relocate multiple times, sometimes over vast distances, in a limited period of time.

Negotiating unfamiliar environments and new transport systems was difficult for some students but offered a sense of independence when achieved. Confronted with social isolation, some students expressed intense loneliness and boredom. One student described not seeking to ‘make friends’ as they did not intend to stay in the location. Some students described experiencing ‘burnout’ towards the end of their placement.

P48 “I think I was starting to get worn out by maybe the four, five-month mark. And I was just like ‘I just want it to be done’.”

#### Theme 3: Optometrist under instruction

The students saw the placement as ideal in that they were able to trial the everyday reality of work within the practice without the obligation of an ongoing commitment or employment contract.

P44 “….you can see for yourself where you’d want to work afterwards. And knowing that’s just temporary, you can last through it. If you don’t like it, it’s okay, it’s going to be just three or six months.”

Overwhelmingly, students were positive about the compulsory aspect of the rural placement, citing that it took them to practice locations many of them would have not otherwise considered. For some, this completely altered their future practice intentions.

P33 “I had no intention of working in a rural setting until I went to (rural location)”

P40 “I grew up in Melbourne and I love Melbourne. I thought I would always want to stay around there but then I went to (rural location) and I was really happy and it’s just so nice to have no traffic lights.”

Despite there being a generally positive attitude towards training in a rural environment, students feared being isolated as a new graduate. They described actively avoiding employment in practices where they would be the sole practitioner, as they were apprehensive about the increased responsibility.

P52 “One of my first questions was ‘Is there anybody else there that will help me?’ and if the answer was no, then that straightaway turned me off going because that’s a big deal—to go from having your hand held at placement, and going to Uni—to your first ever job. So yeah, I would look for a mentor for my first year out.”

The students highlighted the physical presence of a mentor to work alongside as a key element when choosing their future employer. The ability to ask their mentor questions and seek clarification and receive confirmation on clinical judgements was of utmost importance to them as new graduates.

#### Theme 4: Rural practice is more rewarding

Students described the importance of finding a practice and a location that would suit their lifestyle and hobbies. Some students spoke of the desire to practice rurally as if it was a ‘rite of passage’; an important and necessary stage that marks a milestone in their career. They saw it as a chance to seek different experiences, meet new people and challenge themselves professionally.

P39 “I wanted to be thrown into the deep end, you know? The more rural, the better. In those areas, you are valued. You do service quite a few of the surround areas. I wanted to see it all, try it all, meet everyone. It was great”

Many found rural communities more embracing, friendly and relaxed. Others enjoyed the natural surroundings of rural areas. Keen to achieve a work-life balance, students found the concept of rural practice attractive. Many students were attracted to the large remuneration packages being offered in rural locations on graduation. Others described humanitarian reasons for wanting to practice in rural locations, as they felt this was where their clinical skills were most needed. Conversely, some were concerned that there would be nothing of interest to them in a rural community and others were concerned that there would be no work for their partners. For those without partners, they were concerned they would not meet potential suitors. Some students expressed that they were hesitant to accept roles in some rural and remote areas predominantly due to the lack of access to ophthalmology care and support. Overwhelmingly though, the students stated that they wanted to return to a similar location to where they grew up.

P52 “I don’t like big cities because I’m not from one. I did do placement in a big city. But I didn’t like it (the location). I’d rather stay in the country. So because I’m from there, I’m more likely to go back.”

In particular, students who identified as female described how their long-term career aspirations differed from their short-term aspirations. The most common reason cited for this was the desire return home to raise children close to their own family.

P37 “I would like ultimately to move back because this is where I want to raise my kids. I would like my kids to go to the same high school I went to. Grow up in the same area as I did. I feel like it’s a much better environment.”

## Discussion

In accordance with the aim, this study used descriptions of the experience of students who undertook an extended clinical placement, potentially leading to insights pertaining to factors which influence placement success and satisfaction. Reviewing the optometry perspective within the wider LIC literature highlights areas of commonality and also aspects that might be more pertinent to the optometry profession. While data exists for medical and other allied health professions, robust assessment of transferability of experience is necessary. Therefore, this is an important study to have been undertaken because, to date, this is the first instance where Australian optometry students have been placed in community practice for an extended period. Moreover, the optometry profession needs to determine whether such placements are successful before reliance on this placement modality becomes common place.

The familiarity offered by remaining in the same setting for at least 13-weeks meant that students could fully engage in learning opportunities and concentrate on their clinical training [10]. Whether the length of placement undertaken by the participants in this study fosters the same degree of continuity and positive experience as a longer placement, such as those more typical in LICs, is an area further research needs to address. Findings reinforce the concept that extended clinical placements provide students with greater continuity in setting, supervisor and patients [1, 2, 10]. Overall, students reported positive experiences of the extended clinical placement, viewing it as the most important factor in their preparation for professional practice. Transitioning from fledgling trainee optometrist to fully qualified optometrist involved not only the acquisition of knowledge and refinement of clinical skill, but also a shift in identity formation. Participation in clinical care under supervision during clinical placement, and the subsequent evolution of professional identity is a complex area of theoretical study [33, 34]. Yet, it has been argued that the development of professional identity is as important as the development of clinical competencies [35]. The supervisor has been described as playing a critical role in the students formation of identity by ‘providing space, invitation and support’ while also demonstrating a willingness to withdraw from the role of expert-over-novice, and transition to level as colleague-to-colleague [36].

Placements in community settings involve an intricate interplay of learning opportunities fostered through interactions with supervisors, their staff, patients, community members, and potentially other allied health and medical students [17, 37]. This study highlights the complex dynamic between students and their supervisors. Having a positive relationship between student and supervisors has repeatedly been identified as the most important factor in placement satisfaction [38]. Bullying and harassment of students during their clinical training by their supervisors has been widely reported in the nursing literature [39–41]. However, outside the nursing discipline, comparatively little literature exists which has investigated problematic unprofessional behaviours in a student healthcare setting. In Australia, media attention in recent years has illuminated the alleged toxic culture of bullying and harassment in the medical field, illustrating that policy does not set culture [42]. Whether this extends to other Australian health professions has not been reported. A 2014 study, conducted in the United Kingdom, found differences in the manner in which undergraduate medical and nursing students respond to incidents of bullying and harassment [43]. Nursing students were far more likely to respond by challenging the inappropriate behaviour and/or reporting the incident, whereas medical students most often reported doing ‘nothing’ [43]. In the present study, fearful that their supervisor had the power to influence their career, students were reluctant to raise concerns and issues with the University. It appears students perceived their supervisor as a role model who could either enable or constrain their learning and development, as well as their future career opportunities. Furthermore, there is a tendency for students to feel indebted and dependent on the supervisor. Thus, these relationships should be guided and monitored with the utmost care. Placement providers and supervisors must explicitly consider their stance from a student facing lens, recognising that there are always power differentials at play. Failure to take note of power imbalances will likely only encourage a culture where students do not ‘speak up’ with their concerns and instead endure them in silence. Students need to be empowered to highlight concerns when they occur from the outset, with the support of their placement providers and universities, and have these concerns dealt with appropriately.

There is some literature to support the notion that where students have a positive placement experience in a non-metropolitan location, they will be more likely to seek employment in such areas on graduation [44–46]. Students in this study described the rural placement as giving them exposure to a more diverse range of pathologies, the chance to develop independence both personally and professionally, and often more meaningful mentor connections. These findings were similar to those described by Voss et al. [47] in their analysis of student experiences in a South African rural LIC whereby students chronicled that they felt better prepared for practice than their colleagues on more traditional clerkships. This was because they saw a greater number of patients, had a greater diversity in caseload, and exposure to a more complete patient management journey [47]. However, in the present study students lamented that the rural placements came with a greater financial burden and there was potential for isolation to be experienced more strongly as it was less likely students were able to return home at weekends. This study also supports the concept that the longitudinal nature of the placement is of central importance in students developing social and spatial connectedness to the rural community, and ultimately returning to a rural setting to practice [44, 46, 48]. Previous studies have reported that where students are well prepared and adequately supported, immersion in settings of different cultural beliefs and practices leads to valuable learning experiences [37, 49]. Undertaking a rural placement, and actually living within that community for more than a couple of weeks, exposed students to the reality of life in a regional or rural community. Such exposure makes it more likely students will gain an understanding of the impact of health disparities in the local community.

While in general the literature suggests the ‘longer the better’, the ideal length of longitudinal clinical placements is still under debate [11]. The students in this study undertook a 26-week placement (2 × 13-week placements) at a maximum of two community-based sites. Worley et al. [7] reported LICs for medical student with lengths ranging from 6 to 54 weeks, and a median length of 40 weeks. Crampton et al. [50] reasoned 14 weeks is sufficient to allow meaningful mentor connections to be built, while others have identified a ‘turning point’ between 4 and 20 weeks where the cost verse benefit to the student and supervisor begin to balance [51]. The students in this study articulated that the length of the extended placement was appropriate. The duration gave them a chance to develop strong collegial relationships with their co-workers and feel immersed in the practice and community setting in which they were placed. While isolation and loneliness were experienced by a small number of students, the placement length meant students felt they could ‘deal with it’.

Almost all optometry graduates will be employed in community-based practices, thus it is sensible that Universities prepare students sufficiently for this by facilitating placements in community-based settings. However, the long-term sustainability of such placements has not yet been determined in optometry. With increasing student numbers, whether community optometrists will remain willing to provide placements is yet to be seen. Whether there is the potential for practitioners to be reimbursed for their supervisory responsibilities needs to be highlighted with governing bodies.

### Strengths and limitations

A strength of this study is that a breath of honest, rich experiences from participants were described, owing to the quality dialogue between researcher and participant. This undoubtedly increased the information power of this study [24, 52]. However, a limitation is that it was a single-institution study. The current body of literature has explored clinical placements and supervision in ‘discipline silos’ [38]. This study was no different. Inter-professional collaboration would benefit the field and would most certainly address common systemic deficiencies in clinical placements and may go some way to determining whether the ‘rural pipeline concept’ typically seen in medicine is also applicable to other student bodies [20]. With increased collaboration there is a potential for shared learning and problem solving amongst the health disciplines.

### Practice points

Key learning outcomes from the findings of this study have been summarised below in Table 3.

**Table 3:** Practice points for placement success

Expectations	- Universities/placement providers must provide thorough guidelines addressing key expectations specifically such as: roles, rights and responsibilities, key contacts, and key processes - Supervisors and students have a responsibility to adhere to these expectations and discuss their individual expectations with each party on a regular basis to monitor whether they are being met
Professional development and pedagogical training	- Universities/placement providers must ensure supervisors are adequately prepared for clinical teaching especially in the art of providing feedback to students. Training in reflective practice and reflective supervision should be included in programs - Supervisors should engage in training and seek ongoing professional development opportunities. Supervisors have a responsibility to reflect and recognise areas for personal improvement and development
Addressing power differentials	- All parties should acknowledge power imbalances, with particular attention to the fact that students are generally the vulnerable party - Universities/placement providers must provide clear guidelines for reporting incidents. They should provide a separate advocate for students and supervisors, and ensure the advocate periodically engages with each party to monitor whether any issues are present. They must actively encourage the reporting of concerns and follow through on policy guidelines - Supervisors should embrace their role of ‘expert clinician’ while providing a safe learning environment - Students must be empowered with skills, resources and support to identify and respond to inappropriate behaviour
Funding	- Universities/placement providers should seek to provide a level of financial assistance for students to undertake placements, particularly with regard to decreasing the financial barriers associated with rural placements. Where possible they should provide resources and assistance associated with relocation for placement, such as accommodation options

## Conclusion

All students felt that extended clinical placement provided them the opportunity to grow clinically. While the majority of students enjoyed their placement and felt that it was the key component of their training that equipped them for future practice, where the student-supervisor relationship was strained, a high level of anxiety occurred. This was made worse by a perceived lack of university support. Thus, universities and placement providers must provide greater support to students as well as thorough and explicit guidance covering placement expectations for both students and supervisors. Future research should explicitly focus on what support student require during placement, specifically for which student subgroups, and how such supports can be provided effectively. Ultimately systems should be embedded into placement programs to ensure where issues arise, they are dealt with promptly and successfully. Accordingly, it is vital that ongoing professional development and pedagogical training for supervisors underpins continued accreditation. Given students believed they received a richer, more diverse clinical experience during rural placement compared with metropolitan placement, future research should determine firstly whether this is accurate in terms of clinical competencies, and secondly, whether completing a rural placement makes students more likely to practice in a rural location.

## Supplementary Information

**Additional file 1: Appendices 1.** Semi-structured interview guide for focus groups.

## Data Availability

The datasets generated and analysed during the current study are not publicly available due to the sensitive nature of the data and the consent provided for participation but are available from the corresponding author on reasonable request.

## Abbreviation

LIC: Longitudinal Integrated Clerkships
