# Supplementary material for: Student perspectives of extended clinical placements in optometry: a qualitative study
Source: BMC Med Educ. 2022 Jan 25;22:59. doi: 10.1186/s12909-022-03132-0 (PMC8790849; doi:10.1186/s12909-022-03132-0)
Supplement: Supplementary file 1 — Additional file 1: Appendices 1. Semi-structured interview guide for focus groups. [file 12909_2022_3132_MOESM1_ESM.docx]

Appendices 1

**STUDENT PERSPECTIVES OF EXTENDED CLINICAL PLACEMENTS IN OPTOMETRY: A QUALITATIVE STUDY**

Authors

Jacqueline M Kirkman, MOptom, BVisSci^1^

Sharon A Bentley, PhD, MOptom, MPH, BScOptom FACO, FAAO^2^

James A Armitage, PhD, MOptom, BSc (Optom) FACO, FAAO^1^

Ryan J Wood-Bradley, PhD, BSc (Hons)^1^

Craig A Woods, PhD, BSc (Hons) FACO, FAAO^3^

^1^Deakin Optometry, School of Medicine, Deakin University, Waurn Ponds, Australia

^2^School of Optometry and Vision Science, Queensland University of Technology, Kelvin Grove, Australia

^3^School of Optometry and Vision Sciences, University of New South Wales, Sydney, Australia

Corresponding author: Jacqueline Kirkman [jac.kirkman@deakin.edu.au](mailto:jac.kirkman@deakin.edu.au)

**Appendices 1**

**Semi-structured interview guide for focus groups**

How was your extended placement experience?

- What were the positive/negatives of the experience?
- Was it what you thought it would be?
- Did you feel prepared for it?
- How do you think it could have been improved?
  - Reasonable teaching alterations/suggestions?
- How did you rural placement differ from your urban placement?
- Did the placement influence where you wish to work after graduation?

How important is it that students are exposed to rural optometry through placements?

- How do you feel about compulsory rural placements?
- How long do you think rural placements should be and why?
  - What benefits do you see to a short placement (I.e.; 1 to 7 day placements)
  - What benefits do you see to an extended placement (I.e.; 2 to 6 month placements)
  - What differences do you think there are between a short and extended placement?
- Where, in terms of location, did you complete your extended placement and what are the reasons for this?
  - How did you choose to structure your extended placement? (I.e.; did you complete the full 6 months in one practice location or split it into different locations?)
    - What benefits/negatives did you see to this?
- Do you think a placement could altered your future practice intentions?

How interested in working in rural areas are optometry graduates? Elaborate.

- What reasons make students more or less likely to work rurally?
- What incentives would increase student interest in working in rural areas?
- How do you feel about living in a rural area?
- How do you feel about working in a rural area?
- In what way has where you have grown up influenced your future practice intentions?
